# Real-Time PCR in faecal samples of *Triatoma infestans *obtained by xenodiagnosis: proposal for an exogenous internal control

**DOI:** 10.1186/1756-3305-5-59

**Published:** 2012-03-26

**Authors:** Nicolás Bravo, Catalina Muñoz, Nicolás Nazal, Miguel Saavedra, Gabriela Martínez, Eduardo Araya, Werner Apt, Inés Zulantay

**Affiliations:** 1 Faculty of Medicine, Institute of Biomedical Sciences, Cellular and Molecular Biology Program, Basic-Clinical Laboratory of Parasitology, University of Chile, PO 427, Santiago 3, Chile; 2 Gene X-Press, Avenida Sucre 2418, Santiago, Ñuñoa, Chile

**Keywords:** Real-time PCR, Xenodiagnosis, *Triatoma infestans*, Faecal samples, *Trypanosoma cruzi*

## Abstract

**Background:**

The polymerase chain reaction (PCR) has proved to be a sensitive technique to detect *Trypanosoma cruzi *in the chronic phase of Chagas disease, which is characterized by low and fluctuating parasitemia. Another technique proposed for parasitological diagnosis in this phase of infection combines a microscopic search for motile trypomastigote forms in faecal samples (FS) obtained by xenodiagnosis (XD) with conventional PCR (XD-PCR). In this study we evaluate the use of human blood DNA as an exogenous internal control (EIC) for real time PCR (qPCR) combined with XD (XD-qPCR) using chromosome 12 (X12) detection.

**Findings:**

None of the FS-XD evaluated by qPCR amplified for X12. Nevertheless, all the EIC-FS-XD mixtures amplified for X12.

**Conclusions:**

We determined that X12 is useful as an EIC for XD-qPCR because we showed that the FS-XD does not contain human DNA after 30 or more days of XD incubation. This information is relevant for research on *T. cruzi *by XD-qPCR since it allows ruling out inhibition and false negative results due to DNA loss during the process of extraction and purification.

## Findings

The application of the polymerase chain reaction (PCR) has allowed the detection of *Trypanosoma cruzi *in several biological samples obtained from insect vectors and mammalian hosts [1-5]; however, it is a qualitative technique. By contrast, the real-time PCR (qPCR) technique allows detection and quantification of *T. cruzi *parasitic loads, which is particularly important in studies where the effectiveness of chemotherapy is being evaluated [6,7].

Xenodiagnosis (XD) is another non-routine parasitological method, which uses biological vectors to amplify *T. cruzi *[8,9] and has shown good sensitivity and precocity when combined with conventional PCR [10]. The aim of this study is to evaluate the utility of a human DNA fragment as an exogenous internal control (EIC), to be used as a positive control of DNA extraction and as a qPCR inhibitor alert in studies of detection of *T. cruzi *by qPCR in faecal samples (FS) obtained by xenodiagnosis (FS-XD) applied in humans.

### DNA extraction and purification from human blood

All the procedures were carried out in our laboratory. We extracted 5 ml of peripheral blood by venous puncture of an individual serologically negative for Chagas disease, determined by an ELISA Chagas III kit (GrupoBios SA, Chile) and by indirect immunofluorescence IgG (IFI-IgG in-house) tests. The sample was received in 5 ml of guanidine-HCl 6 M EDTA 0.2 M, incubated at 98°C for 15 min in a double boiler and stored at 4°C. Extraction and purification were performed with an initial volume of 200 μl of blood-buffer mixture, according to the indications of the FavorPrep™ Blood Genomic DNA Extraction Mini Kit (Favorgen), with some modifications. We eluted twice with 50 μl of elution buffer that was previously incubated for 10 min at 60°C. Elution volume was 100 μl and samples were stored at -20°C until amplification assays.

### Quantification of human DNA

After extraction and purification, total DNA quantification was carried out using AccuBlue™ High Sensitivity dsDNA Quantitation Kit (Biotium Inc.) and the qPCR instrument Mx3000P (Agilent Technologies) as detector devices. Briefly, the protocol started by mixing the dsDNA Quantitation Solution and the 100x Enhancer Solution in a 100:1 proportion. Then 40 μl of this mixture was loaded in each tube. After this step, 2 μl of DNA sample or 2 μl from each of the eight DNA standards provided by the kit (0, 0.5, 1, 2, 4, 6, 8 and 10 ng/μl dsDNA) were added to each tube. Finally, after mixing and incubating for 5 min in darkness at 25°C, the samples were scanned using the Quantitative Plate Read and the FAM filter of the Mx3000P instrument.

### Obtaining faecal samples of *Triatoma infestans*

Under informed consent approved by the Ethics Committee of the Faculty of Medicine, University of Chile, XD assays were applied using the technique described by Schenone [1999]. Two XD boxes, each containing seven infection-free *T. infestans *nymphs of instars III and IV, were placed for 20-30 min on the forearm of 6 individuals with positive ELISA and IFI-IgG tests for Chagas disease. The boxes were maintained at 27°C and after 30, 60 and 90 days of incubation, the FS of each insect were microscopically examined to detect trypomastigote forms of *T. cruzi*. Thus 3 FS-XD at 30, 60, 90 days were analyzed for each patient (n = 6), resulting in a total of 18 FS-XD. The FS-XD were received in 500 μl PBS buffer pH 7.2, incubated for 15 min at 98°C in a double boiler and centrifuged for 3 min at 3500 rpm. A total of 200 μl of supernatant from each FS-XD was collected and stored at -20°C until use [5].

### Purification of *Triatoma infestans *faecal samples

**Figure 1:**
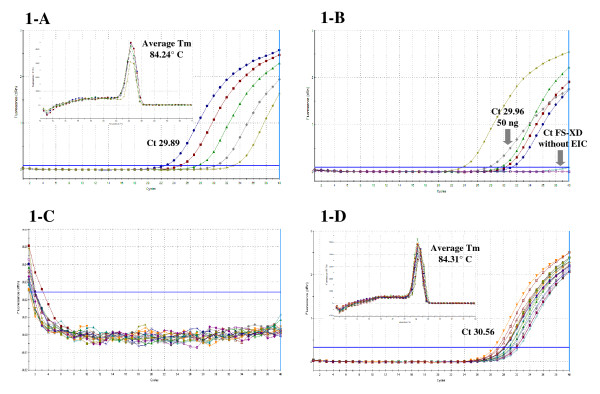
**Amplification and melting curves of real time PCR (qPCR) of faecal samples (FS) of *Triatoma infestans *fed by xenodiagnosis (XD) on patients with chronic Chagas disease, using human blood DNA negative for *T. cruzi *DNA as an exogenous internal control (EIC) by chromosome 12 (X12) detection**. **1-A: **An average Tm of 84.24°C is observed in the melting curve of every 5-fold dilution of human blood DNA negative for *T. cruzi *(EIC). Average Ct value 29.89. X12-qPCR reaction: efficiency = 94.4%, R^2 ^= 0.994 and slope = -3.463. **1-B: **Amplification curve for X12 of 3, 6, 12, 50 and 100 ng of EIC added to FS-XD. The Ct value for 50 ng was 29.96. No Ct value was observed in the FS-XD without EIC. **1-C: **Amplification curve for X12 in purified DNA of 18 FS obtained from XD applied in patients with chronic Chagas disease incubated for 30, 60 and 90 days without EIC, showing absence of human DNA target in the evaluated samples. **1-D: **An average Tm of 84.31°C is observed in the melting curve of 18 FS obtained from XD applied in patients with chronic Chagas disease incubated for 30, 60 and 90 with EIC, indicating that the generated qPCR amplicon matches the Tm value shown in 1-A.

Before the FS-DNA isolating process, we determined the optimal DNA concentration to use as exogenous internal control (EIC). After quantifying DNA extracted from human blood negative for *T. cruzi*, we added direct blood as 3, 6, 12, 50 and 100 ng equivalents of human DNA to DNA of FS free of infection by *T.infestans*. All mixtures were then extracted, purified, and stored at -20°C until amplification. Based on the results obtained in the standardization of qPCR-XD (Figure 1B), we decided to use 50 ng equivalent of negative human blood DNA as an EIC for FS-XD in the study before the DNA isolating process. The 18 *T. infestans *FS-XD without EIC were used to evaluate the presence or absence of human DNA. Also, the EIC (50 ng equivalent of human blood DNA negative for *T. cruzi*) was added to a duplicate of the same samples. The DNA purification of both sets of samples (with and without EIC) was performed using 100 μl of initial volume with the FavorPrep™ Blood Genomic DNA Extraction Mini Kit (Favorgen Biotech Corp.), modified by omitting the step of cell lysis with proteinase K. The elution process was similar to that described for human blood. Samples were maintained at -20°C until qPCR was carried out.

### qPCR protocol for chromosome 12 (X12)

X12 primers were designed using AmplifX v.1.5.4 (N.Jullien) software, and then compared with Nucleotide BLAST (National Library of Medicine) to discount any other unspecific amplification (N. Nazal, personal communication). The N1X12 forward (5'-AGCTGGCTAGACTGTCAT-3') and N2X12 reverse (5'-CTTTGCCGTTGAAGCTTG-3') primers were used at a concentration of 1 μM (Integrated DNA Technologies) and the qPCR reaction was carried out using Brilliant III Ultra-Fast SYBR^® ^Green QPCR Master Mix (Agilent Technologies), Molecular Biology Grade water (Mo Bio Laboratories, Inc.), and 2 μL of DNA isolated from FS-XD with or without EIC, in a final volume of 20 μL. The same qPCR reaction was carried out for 5-fold serial dilutions of DNA extracted from blood of a healthy human mixed with DNA of FS free of infection from *T.infestans*, to estimate the sensitivity and the efficiency of the X12-qPCR reaction and to determine the optimal human blood DNA concentration to use as EIC. The thermal profile included 3 min of pre-incubation at 95°C and 40 amplification cycles (95°C for 5 sec, 65°C for 15 sec and 72°C for 10 sec). The measurement of emitted fluorescence was performed at 72°C at the end of each cycle. After all the amplification cycles a melting curve was run. The results were analyzed with the MxPro v4.1 d (Agilent Technologies) software.

The X12-qPCR reaction for 5-fold serial dilutions of DNA (3, 6, 12, 50 and 100 ng) extracted from human blood negative for *T. cruzi *(EIC) had an efficiency of 94.4%, a coefficient of determination (R^2^) of 0.994 and a slope of -3.463 (data not shown). The amplicon generated had an average melting temperature (Tm) of 84.24°C and a threshold cycle (Ct) average of 29.89 (Figure 1A).

All EIC concentrations were tested on FS-XD and showed amplification of the X12-qPCR reaction. By contrast, no sign of amplification was shown in FS-XD without EIC (Figure 1B).

We decided to use 50 ng equivalent of negative human blood DNA as an EIC for FS-XD before the DNA isolating process, given the fact that this quantity had an early qPCR Ct value without diluting excessively the original FS-XD ((29.96, Figure 1B). Moreover, this Ct value was similar to the average Ct obtained in dilutions of human blood DNA (Figure 1A and 1B).

The 18 FS-XD obtained from 6 individuals with Chagas disease without EIC did not amplify for X12 (Figure 1C). Finally, the 18 EIC + FS-XD mixtures amplified in every case, generating an amplicon with an average Tm of 84.31°C; the average Ct value obtained was 30.56 (Figure 1D).

In the search for an internal control system for XD-qPCR, this study allowed us to verify that triatomines are capable of degrading completely the human DNA they consume within 30 days (Figure 1B and 1C). We designed a system of novel primers using a segment of a DNA sequence which codifies for human X12. The Ct value obtained for the amplification of X12 from 50 ng of DNA added to a sample of non-infected triatomine FS was 29.96 (Figure 1A), and 30.56 with a standard deviation of 1.0011 for the 18 EIC-FS-XD studied, which indicates an optimum recovery of the EIC (Figure 1D). Considering two standard deviations as a criterion, all the Ct values obtained by the amplification of X12 were included in this range. Both the qPCR amplicon generated in the reaction for X12 at EIC-FS-XD and the human blood DNA dilutions were the same, since we obtained a similar Tm value in both assays (Figure 1A and 1C).

These original results are relevant for research on *T. cruzi *by XD-qPCR since they allow ruling out inhibition and false negative results due to DNA loss during the process of extraction and purification. The use of human DNA for the detection of X12 as an EIC of FS obtained by conventional or artificial XD may be useful for the application of FS-qPCR in the parasitological diagnosis of *T. cruzi *and in evaluation of the efficacy of chemotherapeutic treatment of Chagas disease.

Others applications of SYBR Green-based Real Time targeting kinetoplast DNA in vectors of *Leishmania *species (Kinetoplastida, Tripanosomatidae) have been recently described. DNA samples from naturally infected *Lutzomyia *spp. were used as positive controls of reaction for to discriminate among the human pathogenic species representing the most common causative agents of cutaneous and visceral leishmaniasis in Brazilian endemic areas [11].

## Competing interests

The authors declare that they have no competing interests.

## Authors' contributions

NB, CM, MS and IZ formulated the idea; NB, CM, WA and IZ wrote the manuscript; NB, CM, NN, MS, GM and EA performed the experimental procedures. NN, WA and IZ provided critical comments to the protocol and the discussion. All authors approved the final version of this manuscript.
